# Human glucocerebrosidase mediates formation of xylosyl-cholesterol by β-xylosidase and transxylosidase reactions

**DOI:** 10.1194/jlr.RA120001043

**Published:** 2021-01-06

**Authors:** Daphne E. Boer, Mina Mirzaian, Maria J. Ferraz, Kimberley C. Zwiers, Merel V. Baks, Marc D. Hazeu, Roelof Ottenhoff, André R.A. Marques, Rianne Meijer, Jonathan C.P. Roos, Timothy M. Cox, Rolf G. Boot, Navraj Pannu, Herman S. Overkleeft, Marta Artola, Johannes M. Aerts

**Affiliations:** 1 Department of Medical Biochemistry, Leiden Institute of Chemistry, Leiden University, The Netherlands; 2 Department of Medical Biochemistry, Academic Medical Center, Amsterdam, The Netherlands; 3 Department of Medicine, University of Cambridge, Cambridge, United Kingdom; 4 Department of Biophysical Structural Chemistry, Leiden Institute of Chemistry, Leiden University, The Netherlands; 5 Department of Bio-organic Synthesis, Leiden Institute of Chemistry, Leiden University, The Netherlands

**Keywords:** ceramides, cerebrosides, Gaucher disease, glycolipids, inborn errors of metabolism, metabolism, XYLOSYLATION, 25-NBD-cholesterol, 25-[N-[(7-nitro-2-1,3-benzoxadiazol-4-yl) methyl] amino]-27-norcholesterol, 4-MU-β-Glc, 4-methylumbelliferyl-β-D-glucose, 4-MU-β-Xyl, 4-methylumbelliferyl-β-D-xylose, CBE, conduritol-β-epoxide (L-1,2-anhydro-myo-inositol), GalCer, galactosylceramide, GalChol, galactosylated cholesterol, GBA, β-glucocerebrosidase, GCS, glucosylceramide synthase, GD, Gaucher disease, GlcCer, glucosylceramide, GlcChol, 1-O-cholesteryl-β-D-glucoside, NBD C6- ceramide, 6-((N-(7-Nitrobenz-2-Oxa-1,3-Diazol-4-yl)amino)hexanoyl)sphingosine, NPC, Niemann-Pick type C disease, PDB, Protein Data Bank, rhGBA, recombinant human GBA, UDP-Glc, uridine diphosphate glucose, UDP-Xyl, uridine diphosphate xylose, UPLC, ultra performance liquid chromatography, XylCer, xylosylceramide, XylChol, 1-O-cholesteryl-β-D-xylose

## Abstract

Deficiency of glucocerebrosidase (GBA), a lysosomal β-glucosidase, causes Gaucher disease. The enzyme hydrolyzes β-glucosidic substrates and transglucosylates cholesterol to cholesterol-β-glucoside. Here we show that recombinant human GBA also cleaves β-xylosides and transxylosylates cholesterol. The xylosyl-cholesterol formed acts as an acceptor for the subsequent formation of di-xylosyl-cholesterol. Common mutant forms of GBA from patients with Gaucher disease with reduced β-glucosidase activity were similarly impaired in β-xylosidase, transglucosidase, and transxylosidase activities, except for a slightly reduced xylosidase/glucosidase activity ratio of N370S GBA and a slightly reduced transglucosylation/glucosidase activity ratio of D409H GBA. XylChol was found to be reduced in spleen from patients with Gaucher disease. The origin of newly identified XylChol in mouse and human tissues was investigated. Cultured human cells exposed to exogenous β-xylosides generated XylChol in a manner dependent on active lysosomal GBA but not the cytosol-facing β-glucosidase GBA2. We later sought an endogenous β-xyloside acting as donor in transxylosylation reactions, identifying xylosylated ceramide (XylCer) in cells and tissues that serve as donor in the formation of XylChol. UDP-glucosylceramide synthase (GCS) was unable to synthesize XylChol but could catalyze the formation of XylCer. Thus, food-derived β-D-xyloside and XylCer are potential donors for the GBA-mediated formation of XylChol in cells. The enzyme GCS produces XylCer at a low rate. Our findings point to further catalytic versatility of GBA and prompt a systematic exploration of the distribution and role of xylosylated lipids.

The aldopentose xylose resembles the six-membered cyclic pyranose glucose except for lack of the pendant CH_2_OH group. As the main building block of xylan, xylose is a major plant sugar (1). In animals, uridine diphosphate D-xylose (UDP-Xyl) is used by xylosyltransferases to attach a xylose moiety to specific serine or threonine residues in proteoglycans during O-glycosylation (2). Xylose is also found in an O-linked trisaccharide repeat in epidermal growth factor and the notch protein as well as the blood coagulation factors F7 and F9. The human body is unable to synthesize the pentose ring of xylose de novo, but the activated sugar nucleotide UDP-Xyl is obtained from UDP-glucose via UDP-glucuronate generated by UDP-glucuronic acid decarboxylase 1, which is encoded by the *UXS1* gene (3).

Since the first investigations by Fisher and Kent, and Patel and Tappel the structural similarity of xylose to glucose and degradation of β-xylosides in animals has been attributed to β-glucosidases (4, 5). We observed earlier that indeed the lysosomal acid β-glucosidase, also known as glucocerebrosidase (GBA), hydrolyzes 4-methylumbelliferyl-β-xyloside (4-MU-β-Xyl) (Aerts, unpublished observation) in contrast to the nonlysosomal β-glucosidase GBA2 (6). Inherited defects in GBA cause Gaucher disease (GD), a progressive disorder characterized by the accumulation of macrophages loaded with glucosylceramide (GlcCer) in tissues (7, 8). No accumulation of β-D-xylose-containing glycopeptides in patients with GD has been reported, but this possibility has not been formally studied. More recently, another catalytic capacity of GBA has been recognized: the transfer of glucose from β-glucoside substrates to cholesterol, thus generating glucosyl-β-D-cholesterol (GlcChol) (9, 10, 11). Generation of GlcChol by GBA is not merely a test-tube phenomenon; it also takes place in vivo (10). In Niemann-Pick type C disease (NPC), intralysosomal cholesterol is markedly increased owing to genetic defects in either of the two proteins, NPC1 or NPC2, that mediate the egress of the sterol from lysosomes (12). In this pathological condition, GBA actively generates GlcChol (10). Formation of GlcChol can also be induced experimentally by incubating cells with U18666A, an inhibitor of efflux of cholesterol from lysosomes. The transglucosylation reaction in cells is prevented by concomitant inhibition of GBA (10). The β-glucosidase GBA2, tightly associated to the cytoplasmic leaflet of membranes, also exerts transglucosidase activity in vitro and in vivo (10, 13, 14, 15).

The earlier findings on glycon substrate specificity of GBA and the recently noted transglucosidase activity of the enzyme prompted us to examine whether GBA is also able to generate xylosyl-β-D-cholesterol (XylChol). Here we report on the in vitro xylosylation of cholesterol by GBA, generating not only XylChol but notably also di-xylosyl-cholesterol and even small amounts of tri-xylosyl-cholesterol (Xyl_2_Chol and Xyl_3_Chol, respectively). Cells and tissues were found to contain low concentrations of XylChol. Subsequent investigations indicated that GBA2 plays no role in the metabolism (formation of degradation) of xylosylated lipids. Cells when exposed to 4-MU-β-Xyl produce XylChol in an entirely GBA-dependent manner. This reaction is favored during intralysosomal cholesterol accumulation as induced with the agent U88666A. Formation of XylChol also occurs in cells incubated with the plant cyanidin 3-D-xyloside, which is found in berries. Our subsequent studies revealed the presence of xylosylated ceramide (XylCer) in cells and tissues. This lipid is apparently synthesized by glucosylceramide synthase (GCS) using UDP-xylose as a sugar donor. The enzyme is unable to catalyze the formation of XylChol. Our findings on the unexpected existence of xylosylated lipids and their metabolism by glucocerebrosidase are discussed in relation to Gaucher disease.

## Materials and methods

### Materials

25-[N-[(7-nitro-2-1,3-benzoxadiazol-4-yl)methyl]amino]-27-norcholesterol (25-NBDcholesterol) and ceramide d18:1/18:1 was purchased from Avanti Polar Lipids (Alabaster, AL). (6-((N-(7-Nitrobenz-2-Oxa-1,3-Diazol-4-yl)amino)hexanoyl)sphingosine) (NBD C6-Ceramide) was purchased from Invitrogen (Waltham, MA). 4-Methylumbelliferyl β-D-glucoside (4-MU-Glc) and 4-methylumbelliferyl β-D-xyloside (4-MU-Xyl) were purchased from Glycosynth™ (Cheshire, United Kingdom). Cyanidin-3-O-β-D-xyloside was obtained from Toronto Research Chemicals (North York, Canada). Uridine diphosphate glucose (UDPGlc), cholesterol, cholesterol trafficking inhibitor U18666A, 1-O-cholesteryl-β-D-glucose (βcholesteryl glucose, β-GlcChol), and ammonium formate (LC-MS quality) were from Sigma-Aldrich (St Louis, MO). Uridine diphospho-α-D-xylopyranoside (UDP-Xyl) was purchased from CarboSource Services (Athens, Supported in part by Grant #DE-FG02- 93ER20097). GBA inhibitor Conduritol-β-epoxide (L-1,2-anhydro-myo-inositol; CBE) was purchased from Enzo Life Sciences Inc. (Farmingdale, NY), GBA inhibitor ME656 (16) GBA2 inhibitor N-(5-adamantane-1-yl-methoxy-pentyl)-deoxynojirimycin (AMP-DNM) (17), D-xylo-cyclophellitol, ceramide d17:0/16:0, and ^13^C_6_-GlcChol were synthesized at Leiden Institute of Chemistry (Leiden, The Netherlands) (10, 18, 19). Cerezyme®, a recombinant human GBA (rhGBA) was obtained from Genzyme (Genzyme Nederland, Naarden, The Netherlands). LC-MS-grade methanol, 2-propanol, water, and HPLC-grade chloroform were purchased from Biosolve. XylChol was synthesized as described in supplemental materials and methods.

### Collection of Niemann-Pick type C mouse livers and GD patient spleens

Livers from Npc1^−/−^ mice (Npc1^nih^), along with wild-type littermates (Npc1^+/+^), were collected in a previous study (10). All human spleens were obtained with consent at the Academic Medical Center in Amsterdam following therapeutic splenectomy. The phenotype of the subjects was established by clinical examination. All organs were stored at −80°C. Later, homogenates were made from the frozen material in water.

### Cloning and expression of cDNAs encoding GBA2, GBA3, and UGCG (GCS)

The design of cloning primers was based on NCBI reference sequences NM_020944.2 for human GBA2 and NM_020973.3 for human GBA3. HeK293 cell lines were generated with overexpressed GBA2 or GBA3 as described previously (10). GCS in HeLa cells was knocked down by the CRISPR-Cas9 system (20). For overexpression, the coding sequence of UGCG (GCS) was amplified by PCR (using the following oligonucleotides: sense. 5′- GGGGACAAGTTTGTACAAAAAAGCAGGCTACCACCATGGCGCTGCTGGACCTG-3′ and antisense 5′-GGGGACCACTTTGTACAAGAAAGCTGGGTCTTATACATCTAGGATTTCCTCTG-3′) and cloned into pDNOR-221 and subcloned in pcDNA3.1-Zeo via Gateway cloning system (Invitrogen). Correctness of the construct was verified by sequencing. HEK293 cells were obtained from the “American Type Culture Collection” and cultured in Iscove's modified Dulbecco's medium with 5% FBS and penicillin/streptomycin under 5% CO2 at 37°C. For transfection, cells were seeded at 75% confluence in 6-well plates and transfected using PEI Transfection reagent (Polysciences Inc., Warrington, FL) according to the manufacturer's instructions, at a PEI:DNA ratio of 3:1.5.

### Culturing and collection of GD fibroblasts

The fundamental research with cell lines was conducted with the approval of the Ethical Committee of the Academic Medical Centre. Control and GD patient fibroblasts homozygous for N370S, L444P, and D409H mutations in GBA were cultured in HAMF12-DMEM medium supplied with 10% FBS and penicillin/streptomycin at 37°C under 7% CO_2_. Fibroblasts cultured from patients with the unique cardiovascular form of Gaucher disease were kindly provided by Dr Amparo Chabás (Institut de Bioquimica Clinica, Corporacio Sanitaria, Barcelona, Spain) and by Professor Ari Zimran (Sha'are Zedek Hospital Medical Centre, Jerusalem, Israel). Fibroblasts were collected by trypsinization followed by 3× washing with ice-cold PBS. Cells were homogenized in 25 mM potassium phosphate buffer pH 6.5 supplemented with 0.1% (v/v) Triton X-100 by sonication on ice.

### In vitro assays with fluorogenic 4-methylumbelliferyl-β-D-glycosides

Enzymatic activity of GBA was measured with 3.7 mM 4-MU-β-Glc or 4-MU-β-Xyl, dissolved in 150 mM McIlvaine buffer (pH 5.2 supplemented with 0.2% [w/v] sodium taurocholate, 0.1% [v/v] Triton X-100, and 0.1% [w/v] BSA) (21). The reaction was stopped with NaOH-glycine (pH 10.3), and fluorescence was measured with a fluorimeter LS-55 (Perkin-Elmer, Beaconsfield, United Kingdom) at λ_ex_ 366 nm and λ_em_ 445 nm. The enzymatic activity of GBA2 was measured in lysates of cells overexpressing the enzyme using the same conditions as above but without the presence of detergents and at pH 5.8. The enzymatic activity of GBA3 was measured in the absence of detergents in 100 mM Hepes buffer at pH 7.0 (22). Stimulation of GBA activity by the activator protein saposin C, produced recombinantly in *Escherichia coli* (23), was monitored with 3.7 mM 4-MU-β-Glc as the substrate in 150 mM McIlvaine buffer pH 4.5 containing 0.1% (w/v) BSA and 0.4 mg/ml phosphatidylserine (24).

### In vitro assay of transglycosidase activity with fluorescent 25-NBD-cholesterol as acceptor

Recombinant GBA and lysates of HEK293 cells overexpressing GBA2 and GBA3 were used to determine transglycosidase activity of each enzyme. The assays were performed as described earlier (10). First, lysates overexpressing GBA2 or GBA3 were preincubated with 5 μM CBE for 20 min (samples containing diluted recombinant GBA were preincubated in the absence of CBE). To each of the samples the appropriate buffer containing 4-MU-Xyl or 4-MU-Glc was added for a final donor concentration of 3 mM and a final concentration of 40 μM 25-NBD-cholesterol as acceptor. Transglycosidase activity of GBA2-overexpressing cells was measured in a 150 mM McIlvaine buffer pH 5.8, and the assay for recombinant GBA was done in a 150 mM McIlvaine buffer pH 5.2 containing 0.1% BSA, 0.1% Triton X-100, and 0.2% sodium taurocholate. For GBA3 the assay contained 100 mM Hepes buffer, pH 7.0. The reaction was terminated by addition of chloroform/methanol (1:1, v/v) and lipids were extracted according to Bligh and Dyer (25). Thereafter, lipids were separated by thin layer chromatography on HPTLC silica gel 60 plates (Merck, Darmstadt, Germany) using chloroform/methanol (85:15, v/v) as eluent followed by detection of NBD-labeled lipids using a Typhoon Variable Mode Imager (GE Healthcare Bio-Science Corp., Piscataway, NJ) (6).

### In vitro assay of GCS activity with fluorescent NBD C6-ceramide as acceptor

HeLa, HeLa GCS KO, and HeLa GCS KO with overexpression of GCS cells were homogenized in 100 mM potassium phosphate buffer pH 7.5 supplemented with 4 mM MgCl_2_ by sonication on ice. These homogenates were incubated with 35 μM NBD C6-ceramide and 10 mM of UDP-Glc or UDP-Xyl for 16 h. Lipids were extracted, separated, and visualized as described above.

### In vitro assay of transglycosidase activity with cholesterol as acceptor

Assays with natural cholesterol or ceramide d18:1/18:1 as acceptor were performed exactly as described in the sections above and the subsequent analysis of products was performed by LC-MS/MS as described in the section below. In brief for cholesterol: pure rhGBA was incubated at 37°C with a final concentration 6 of 32 μM cholesterol and 3.0 mM 4-MU-β-Xyl or 4-MU-β-Glc in 150 mM McIlvaine buffer pH 5.2 containing 0.1% BSA, 0.1% Triton X-100, and 0.2% sodium taurocholate for the indicated time periods. In short for ceramide: 10 mM of UDP-Glc or UDP-Xyl was incubated with 0.16 μM ceramide d18:1/18:1 for 16 h. All the incubations were stopped by addition of chloroform/methanol (1:1, v/v), and lipids were extracted according to Bligh and Dyer (25).

### Assays with cultured RAW264.7 and HEK293 cells

Experiments with cultured RAW264.7 and HEK293 cells exposed to 3.7 mM 4-MU-β-Xyl or 3.7 mM cyanidin-3-O-β-D-xyloside in the medium, either with or without preincubation in the presence of U18666A (10 μM), inducing lysosomal cholesterol accumulation, were performed as described earlier (10). Lysosomal GBA was irreversibly inhibited by prior incubation of cells with 300 μM CBE or 20 nM ME656 (16). Cells were harvested and lipids extracted as described earlier (10).

### Synthesis of xylosylated cholesterol

The synthesis of β-cholesteryl xyloside is described in the supplemental materials and methods.

### Generation of XylCer

XylCer was enzymatically generated by incubation of rhGBA with a final concentration of 4 μM ceramide d18:1/18:1 and 3.4 mM 4-MU-β-Xyl dissolved in 150 mM McIlvaine buffer (pH 5.2 supplemented with 0.2% [w/v] sodium taurocholate, 0.1% [v/v] Triton X-100, and 0.1% [w/v] BSA) for 16 h at 37°C. Lipids were extracted according to Bligh and Dyer (25) and dissolved in MeOH for HPTLC and LC-MS/MS measurement.

### In vitro assays of glucosylceramide synthase

HeLa cells, HeLa cells overexpressing GCS, HeLa GCS KO cells, MEB-4, and GM95 fibroblasts were homogenized in 100 mM potassium phosphate pH 7.5 containing 4 mM MgCl_2_. For HPTLC analysis homogenates were incubated with a final concentration of 0.2 mM NBD-C6-Ceramide and 20 mM UDP-Glc or UDP-Xyl in 100 mM KpI pH 7.5 containing 4 mM MgCl_2_ for 16 h at 37°C. For LC-MS/MS analysis cell homogenates were incubated with 35 μM ceramide d18:1/18:1 and 20 mM UDP-Glc or UDP-Xyl in 100 mM KpI pH 7.5 containing 4 mM MgCl_2_ for 16 h at 37°C.

### LC-MS/MS analysis

A Waters Xevo-TQS micro instrument was used in all experiments. The instrument consisted of an ultra performance liquid chromatography (UPLC) system combined with a tandem quadruple mass spectrometer as mass analyzer. Data were analyzed with Masslynx 4.1 Software (Waters, Milford, MA). Tuning conditions for GlcChol, XylChol, GlcSph, and XylSph in ES+ (electrospray positive) mode are presented in supplemental Table 2. During this study, all lipids were separated using an Acquity BEH C18 reversed-phase column (2.1 × 50 mm, particle size 1.7 μm; Waters). The column temperature and the temperature of the auto sampler were kept at 23 and 10°C, respectively during the run. The flow rate was 0.250 ml/min and volume of injection 10 μl.

### Analysis of GlcChol and XylChol by LC-MS/MS

For the identification of XylChol, the extracted sample was dried and dissolved in methanol. MS parents scan and daughters scan were performed. As for GlcChol (10), the most abundant species of XylChol are ammonium adducts, [M+NH4] +, and the product ion 369.3 represents the cholesterol part of the molecule after loss of the xylose moiety. Ammonium adducts of XylChol, Xyl_2_Chol, and Xyl_3_Chol showed the transitions 536.5 > 369.3, 668.5 > 369.3, and 800.5 > 369.3, respectively. The transitions for GlcChol are 566.5 > 369.3 and for ^13^C_6_-GlcChol 572.5 > 369.3.

For multiple reaction monitoring an isocratic UPLC program was applied during 5.5 min consisting of 10% eluent A (2-propanol:H_2_O 90:10 [v/v] containing 10 mM ammonium formate) and 90% eluent B (methanol containing 10 mM ammonium formate). The divert valve of the mass spectrometer was programmed to discard the UPLC effluent before (0–0.8 min) and after (4.5–5.5 min) the elution of the analytes to prevent system contamination. The retention time of both GlcChol and the internal standard ^13^C_6_-GlcChol was 1.36 min. XylChols were either synthesized or generated in vitro by incubation of GBA with 4-MU-β-Xyl and cholesterol. The retention time of XylChol, Xyl_2_Chol, and Xyl_3_Chol was 1.71, 1.49, and 1.40 min, respectively (supplemental Fig. 1). Finally, 50 μl plasma was spiked with pure XylChol (concentrations: 0, 0.5, 1, 2.5, 5, 10, 25, 50, 100, 200, 500, 1,000 pmol XylChol/ml of plasma), an internal standard ^13^C_6_-GlcChol was added, and samples were extracted. A linear response was obtained over the entire concentration range (*y* = 0.0108*x* + 1.9888, R^2^ = 0.998). The area from transition XylChol over the area from the transition of internal standard (the ratio) was plotted against the concentration of XylChol in the plasma samples. The limit of detection was 0.5 pmol/ml plasma with a signal-to-noise ratio of three and the limit of quantification was 1.6 pmol/ml plasma with a signal-to-noise ratio of 10. Calculation of the signal-to-noise ratio was done using the peak-to-peak method.

### LC-MS/MS quantification of GlcChol and XylChol produced in vitro

Following incubation of rhGBA and cholesterol with either 4-MU-β-Glc or 4-MU-β-Xyl, lipids were extracted according to the method of Bligh and Dyer by addition of methanol, chloroform, and water (1:1:0.9, v/v/v). The lower phase was taken to dryness in an Eppendorf concentrator. Isolated lipids were purified by water/butanol extraction (1:1, v/v). The upper phase (butanol phase) was dried and dissolved in methanol and sonicated in a bath sonicator, and samples were analyzed by LC-MS.

### LC-MS/MS quantification of GlcChol and XylChols in cultured cells and organs

Cells were homogenized in 25 mM potassium phosphate buffer pH 6.5 containing 0.1% (v/v) Triton, by sonication on ice. Livers and spleens were homogenized in water. Prior to extraction, ^13^C-labeled GlcChol and ceramide d17:0/16:0 in methanol (both used as an internal standard) were added to the homogenate. Samples were then treated with methanol:chloroform (1:1, v/v) to precipitate the proteins, following further extraction of the supernatant as described above.

### Analysis of GlcCer and XylCer by LC-MS/MS

To measure GlcCer and XylCer, lipids were deacylated (26) after Bligh and Dyer extraction with methanol, chloroform, and 100 mM formate buffer pH 3.1 (1:1:0.9, v/v/v). Next, the sample was evaporated and purified by water/butanol extraction (1:1, v/v). The butanol phase was dried, the final sample was dissolved in methanol, and GlcSph and XylSph levels were measured. GlcSph was analyzed as published previously (19, 27). For XylSph identification the sample was introduced in the mass spectrometer using LC-MS/MS (from 0 to 6.5 min to the detector), using eluent A (H_2_O:formic acid 99.5/0.5 [v/v] containing 1 mM ammonium formate) and eluent B (methanol:formic acid 99.5/0.5 [v/v] containing 1 mM ammonium formate). A mobile phase gradient was used during the run: 0.00 min 0% B, 2.50 min 100% B, 6.00 min 100% B, 6.05 min 0% B, and 6.50 min 0% B. MS parents scan and daughters scan were performed (supplemental Fig. 2). The multiple reaction monitoring of precursor > fragment ions (*m/z* GlcSph 462.3 > 282.3 and XylSph 432.7 > 282.3) was used for quantification.

### Docking

Protein Data Bank (PDB) 2V3D was obtained from the PDB database and the N-butyl-deoxynojirimycin ligand was removed. XylChol was made with JLigand (28) and manually docked using COOT (29). Inhibitor 8 crystal structures of PDB 2V3D, 2XWE, and 6Q6L were used as reference structures during docking (16, 30, 31). Final figures were made using CCP4MG (32).

### Protein determination

Protein was measured with the Pierce BCA Protein Assay kit (Thermo Scientific). Absorbance was measured in EL808 Ultra Microplate Reader (BIO-TEK Instruments Inc.) at 562 nm.

### Statistical analysis

Values in figures are presented as a mean ± SD. Data were analyzed by unpaired Student's *t* test or Mann-Whitney U test. *P* values < 0.05 were considered significant. ∗*P* < 0.05, ∗∗*P* < 0.01, and ∗∗∗*P* < 0.001.

## Results

### Cleavage of 4-methylumbelliferyl-β-D-xylose by GBA

**Fig. 1:**
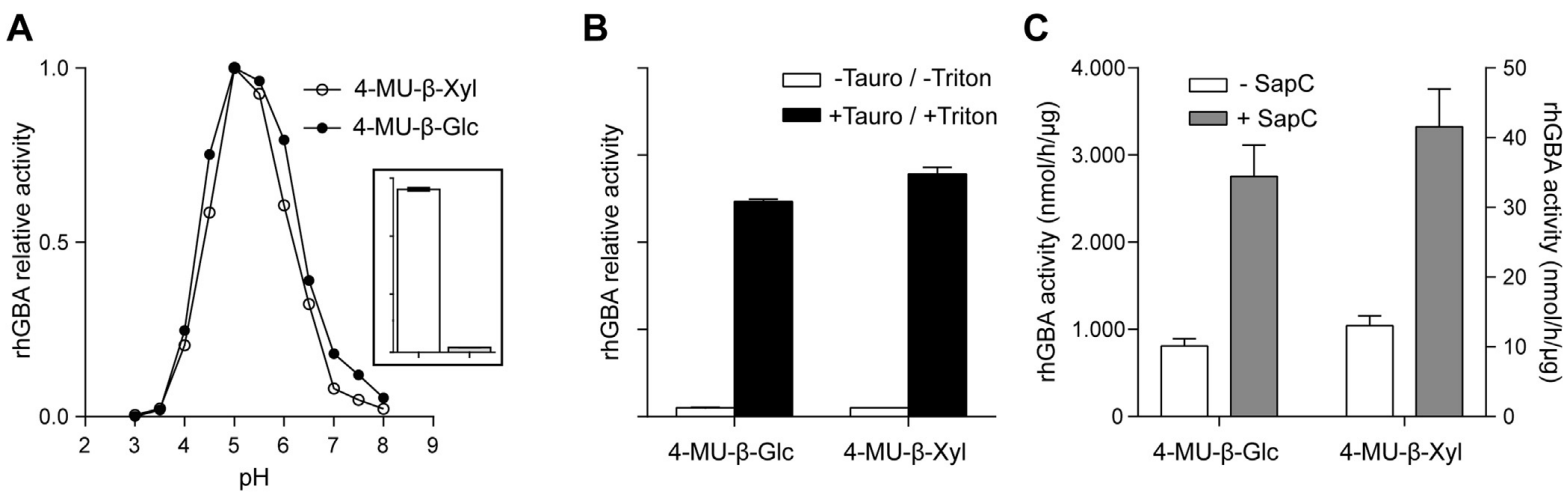
Cleavage of 4-MU-β-Glc and 4-MU-β-Xyl by recombinant human rhGBA. A: pH optimum of 4-MU release by GBA from 4-MU-β-Glc (closed circles) and 4-MU-β-Xyl (open circles). Inset: specific activity of rhGBA with 4-MU-β-Glc and 4-MU-β-Xyl. B: Stimulation by 0.2% (w/v) taurocholate of 4-MU release from the substrates 4-MU-β-Glc (left) and 4-MU-β-Xyl (right) at pH 5.2 in the presence of 0.1% (v/v) Triton X-100 and 0.1% bovine serum albumin. Expressed as 100% is the activity in the absence of taurocholate. C: Stimulation of 4-MU release from the substrates 4-MU-β-Glc (left axis) and 4-MU-β-Xyl (right axis) by recombinant saposin C at pH 4.5 in the presence of phosphatidylserine. (n = 3, mean ± SD).

We first compared the ability of pure rhGBA to cleave 4-MU-β-Xyl and 4-MU-β-Glc. The enzyme releases fluorescent 4-MU from both substrates, but the noted activity toward 4-MU-β-Xyl is around 50-fold lower as the result of a higher *K*_m_ and lower *V*_max_ (Fig. 1A inset, supplemental Table S1). The activity of GBA toward both substrates shows a similar pH optimum (Fig. 1A) and taurocholate stimulation (Fig. 1B). The stimulatory effect of recombinantly produced saposin C on GBA-mediated cleavage of 4-MU-β-Glc and 4-MU-β-Xyl is also comparable (Fig. 1C). The *k*_cat_/*K*_m_ of recombinant GBA is about 40-fold higher for 4-MU-β-Glc than 4-MU-β-Xyl (Table S1). The retaining β-glucosidase GBA employs the Koshland double displacement mechanism for catalysis with E340 as nucleophile and E325 as acid/base (33). Blocking glutamate E340 by covalent inhibition of cyclophellitol abolishes the activity of GBA (34). Of note, the activity of GBA toward 4-MU-β-Glc and 4-MU-βXyl substrates was found to be quite comparably inhibited during preincubation with cyclophellitol for 90 min. The slightly higher apparent IC50 observed with 4-MU-β-Glc (85 nM) than with 4-MU-β-Xyl (61 nM) is likely explained by the greater protection by the β-D-glucose substrate against irreversible inhibition of GBA. Recently, a xylose analogue of cyclophellitol was synthesized (18). It was observed that GBA is also irreversibly inactivated by D-xylo-cyclophellitol, although with lower affinity than cyclophellitol (supplemental Table S1). Again, the apparent IC_50_ determined with 4-MUβ-Glc (10.2 μM) is slightly higher than with 4-MU-β-Xyl substrate (6.4 μM), presumably owing to better protection of GBA against irreversible inhibition by the presence of 4-MU-β-Glc.

### Transxylosylation activity of GBA

**Fig. 2:**
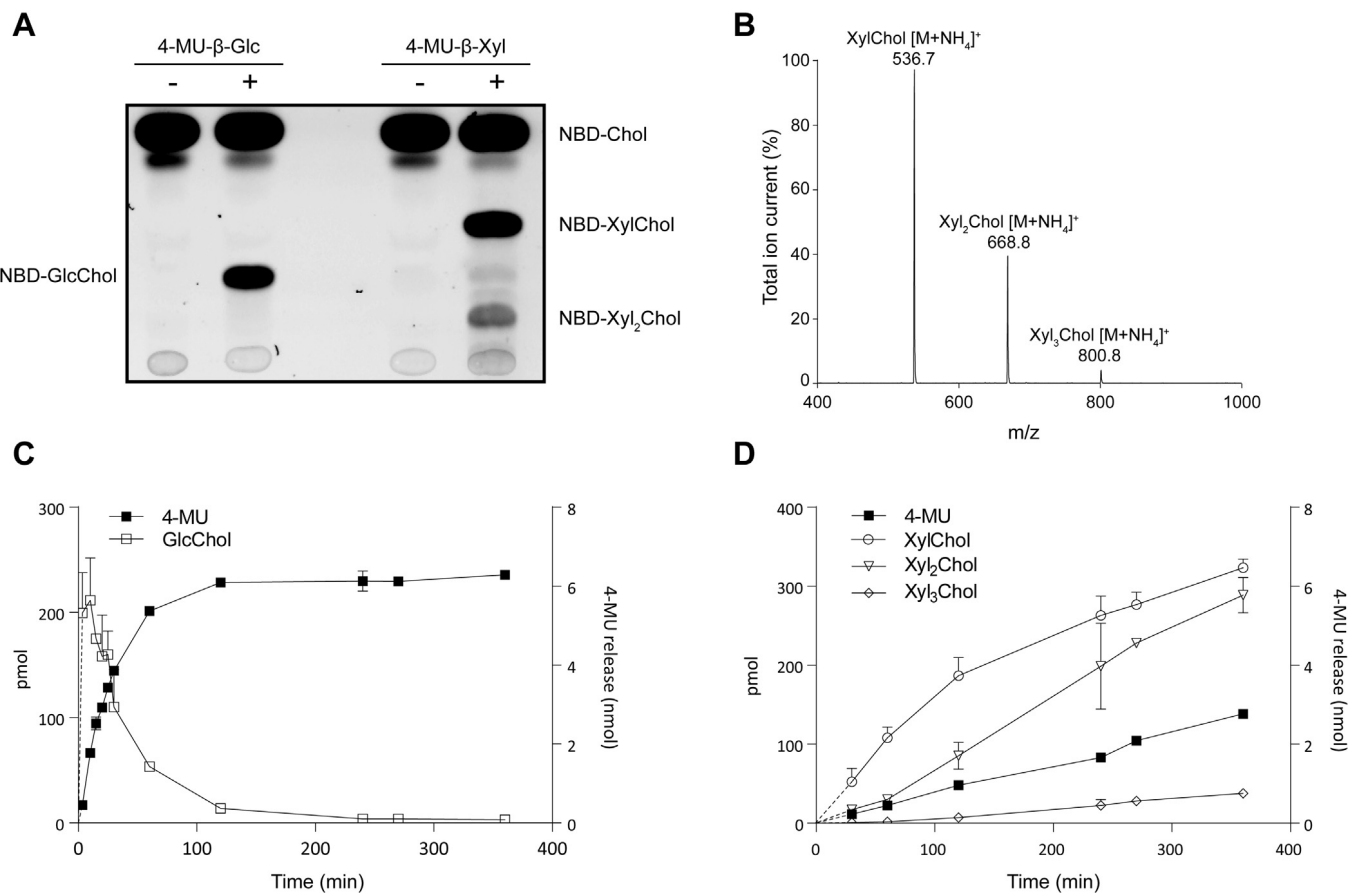
Transxylosylation and transglucosylation of cholesterol by GBA. A: HPTLC analysis of fluorescent products formed from 25-NBD-cholesterol following incubation with rhGBA in the presence of 4-MU-β-Glc or 4-MU-β-Xyl for 16 h. B: LC-MS/MS analysis of products formed after 1 h incubation of rhGBA, cholesterol, and 4-MU-β-Xyl. C: Release of 4-MU from 4-MU-β-Glc and concomitant formation of glucosylated cholesterol in time. D: Release of 4-MU from 4-MU-β-Xyl and concomitant formation of xylosylated cholesterol in time. rhGBA was incubated at pH 5.2 in the presence of taurocholate and Triton X-100 with 3.7 mM 4-MU-substrates for indicated times (n = 2, mean ± SD).

We investigated rhGBA with respect to transxylosylation activity. For this, rhGBA was incubated for 16 h with 4-MU-β-Glc or 4-MU-β-Xyl as donor and fluorescent 25-NBD-cholesterol as acceptor, and the resulting products were analyzed by HPTLC and fluorescence scanning. Formation of fluorescent sterol metabolites occurred with both donors (Fig. 2A). With 4-MU-β-Glc, glucosylated 25-NBD-cholesterol is formed as described earlier (10). With 4-MU-β-Xyl, two novel fluorescent metabolites were detected, presumed to be mono- and di-xylosylated 25-NBD-cholesterol (Fig. 2A). Next, we used natural cholesterol as the acceptor and 4-MU-β-Xyl as the donor. After incubation with rhGBA with pure 4MU-β-Xyl, the formed products were analyzed by LC-MS/MS. Formation of XylChol, Xyl_2_Chol, and traces of Xyl_3_Chol was detected (Fig. 2B). Apparently, sequential transxylosylation can occur. In sharp contrast, incubation of GBA and cholesterol with 4-MU-β-Glc only renders GlcChol as the product (Fig. 2C) (10).

### Time dependence of glycosidase and transglycosidase activities of GBA

GBA and cholesterol were incubated with 4-MU-β-Glc or 4-MU-β-Xyl at 37°C for different time periods. The release of 4-MU and formation of glycosylated products were determined. The formed GlcChol was already maximal after 30 min and subsequently declined with time (Fig. 2C). Apparently, the formed GlcChol is subject to subsequent hydrolysis by GBA. In sharp contrast, XylChol showed no prominent reduction over time, and Xyl_2_Chol was formed after a lag period (Fig. 2D). This suggests that XylChol is hardly hydrolyzed and acts as acceptor for further xylosylation. This process continues with Xyl_2_Chol acting as acceptor rendering Xyl_3_Chol (Fig. 2D). A comparison of the release of 4-MU with concomitant formation of glycosylated sterol indicates that GBA shows considerably higher net transxylosylation than transglucosylation (Fig. 2).

### In vivo formation of xylosylated cholesterol

**Fig. 3:**
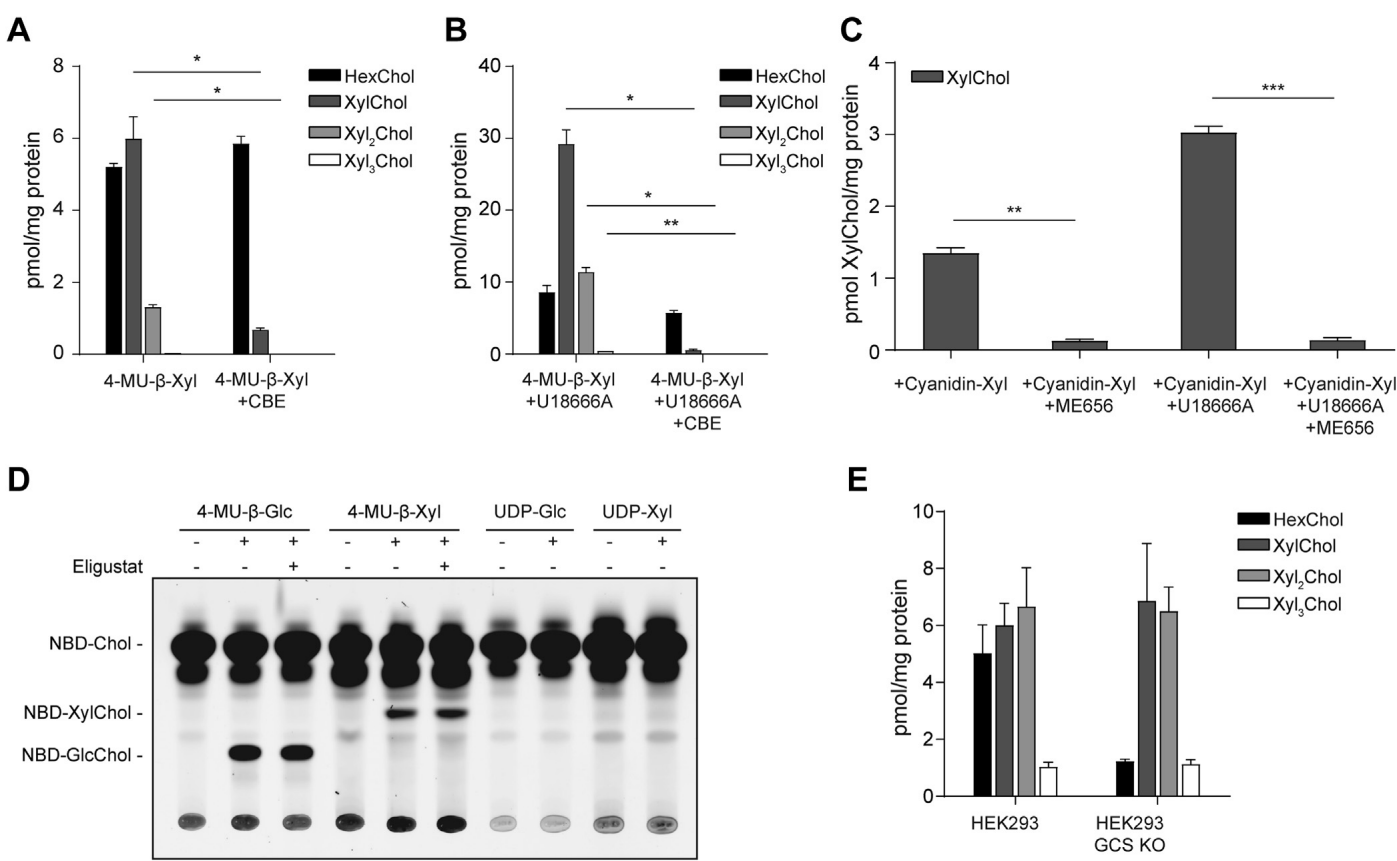
Formation of xylosylated cholesterols. A: LC-MS/MS detection of formed XylChol and HexChol (GlcChol or GalChol) in RAW264.7 cells incubated with 3.7 mM 4-MU-β-Xyl for 24 h in the presence/absence of CBE (n = 3 technical replicates ± SD). B: Experiment A in the presence of 10 μM U18666A. C: LC-MS/MS detection of formed xylosylated cholesterol in RAW264.7 cells incubated with 3.7 mM cyanidin-3-O-β-D-xyloside for 24 h in the presence/absence of U18666A with or without GBA inhibitor ME656 (n = 2 technical replicates ± SD). D: HPTLC of in vitro formation of xylosylated cholesterols by HeLa cell lysate using NBDChol as acceptor and 4-MU-Glc, 4-MU-Xyl, UDP-Glc, and UDP-Xyl as (potential) sugar donors in the presence/absence of GCS inhibitor Eligustat. E: LC-MS/MS detection of formed xylosylated and glucosylated cholesterols in HEK293 and HEK293 GCS KO cells exposed to 3.7 mM 4-MU-β-Xyl and U18666A for 24 h (n = 3 technical replicates ± SD). ∗*P* < 0.05; ∗∗*P* < 0.01; ∗∗∗*P* < 0.001.

To substantiate our in vitro findings, potential transxylosylation by cultured RAW264.7 cells exposed to 3.7 mM 4-MU-β-Xyl was investigated. The cells were incubated with or without the irreversible GBA inhibitor conduritol B-epoxide (CBE) either in the absence (Fig. 3A) or presence of 10 μM U18666A (Fig. 3B) to induce lysosomal accumulation of cholesterol (10). Formation of XylChol, Xyl_2_Chol, and Xyl_3_Chol was detected by LC-MS/MS. The levels of xylosylated cholesterols were markedly increased by the exposure of cells to U18666A, and formation was prohibited by prior inhibition of GBA with CBE (Fig. 3A, B).

Several β-xylosidic compounds are known to be produced by plants, and these compounds are likely to be assimilated after ingestion of food (35, 36). We therefore investigated whether cyanidin-3-O-β-D-xyloside from berries (see supplemental Fig. S3 for chemical structure) can act as a sugar donor in cellular formation of XylChol. For this purpose, RAW264.7 cells were incubated with cyanidin-3-O-β-D-xyloside for 24 h and the formation of XylChol was monitored. Indeed, xylosylated cholesterol was formed by the RAW264.7 cells in a GBA-dependent manner, as indicated by the inhibitory effect of GBA inactivation with the specific irreversible inhibitor ME656 (Fig. 3C, see supplemental Fig. S3 for ME656 chemical structure). We next investigated the possible involvement of the enzyme GCS (37, 38). No involvement of GCS in the synthesis of XylChol was detected with an in vitro GCS assay (Fig. 3D). Furthermore, HeLa cells in which GCS was inactivated by CRISPR-Cas9 were found to produce XylChol on par with the corresponding normal cells when exposed to 4-MU-β-Xyl and U18666A (Fig. 3E).

### Specificity of transxylosylation

We next studied the transxylosylation ability of two other human retaining β-glucosidases, GBA2 and GBA3. We earlier noticed that GBA2, but not GBA3, mediates the transfer of the glucosyl moiety from 4-MU-β-D-Glc to cholesterol or ceramide (10). Although this finding was recapitulated (supplemental Fig. S4), concomitantly no xylosylation by GBA2 was detectable. This finding is consistent with the noted inability of GBA2 to hydrolyze 4-MU-β-Xyl (6). In contrast, GBA3, albeit less prominent than GBA, was found to be able to hydrolyze 4-MU-β-Xyl as well as to transxylosylate cholesterol (supplemental Fig. S4).

### β-Xylosidase and transxylosylation activities of mutant glucocerebrosidase of patients with GD

Mutant forms of GBA commonly encountered in patients with GD were studied regarding catalytic features. As source of GBA enzymes, lysates of control and GD patient fibroblasts homozygous for N370S, L444P, and D409H mutations in GBA were used. Cell lysates were incubated with either 4-MU-Glc or 4-MU-Xyl to determine their glucosidase and xylosidase activity by measuring the 4-MU release. Lysates were also incubated with 4-MU-Glc and 4-MU-Xyl concurrently with cholesterol as the acceptor, followed by GlcChol and XylChol measurement (Table 1). Of note, we related various activities to β-glucosidase activity. It should be kept in mind that, in lysates of GD patient fibroblasts, the absolute β-glucosidase activity is reduced compared with lysates of fibroblasts from healthy individuals. In the case of most patient fibroblast lysates the β-glucosidase and β-xylosidase were reduced commensurately; thus, the mutant enzyme showed no preference for one or the other substrate. Likewise, transglucosidase and transxylosidase activity levels in patient lysates were changed in proportion to their β-glucosidase activity. However, in the case of N370S GBA, there was a subtle decrease in the xylosidase/glucosidase ratio compared with the control enzyme. In addition, we consistently observed a reduced transglucosylation/glucosidase activity in GD fibroblasts with a D409H mutation in GBA compared with control cells. This was noted with multiple patient cell lines. Similar observations were made with mutant GBA enzymes expressed in HEK293T cells lacking endogenous GBA following CRISPR-Cas9-mediated gene disruption, and GBA expressed in GD fibroblasts with virtually no endogenous GBA (data not shown). Thus, only minor abnormalities in the various catalytic activities of mutant GBA enzymes were noted.

**Table 1:** Catalytic features of mutant GBA enzymes

Mutation	Xylosidase/glucosidase	Transglucosylation/glucosidase	Transxylosylation/glucosidase	Transxylosylation/xylosidase
Ctrl	0.035 ± 0.007	0.654 ± 0.076	0.044 ± 0.006	1.248 ± 0.157
N370S	0.018 ± 0.001	0.391 ± 0.081	0.029 ± 0.012	1.615 ± 0.652
L444P	0.028 ± 0.000	0.571 ± 0.066	0.044 ± 0.004	1.557 ± 0.149
D409H	0.028 ± 0.004	0.182 ± 0.071^a^	0.030 ± 0.001	1.057 ± 0.038

Relative enzymatic activities in fibroblasts lysates from patients with GD incubated with 4-MU-Glc or 4-MU-Xyl to determine glucosidase and xylosidase activity, and values are expressed ± SD. Assays were performed with lysates from at least two distinct patients (*n* = 2).

a *P* < 0.05 compared with control.

### Natural occurrence of xylosylated cholesterol

**Fig. 4:**
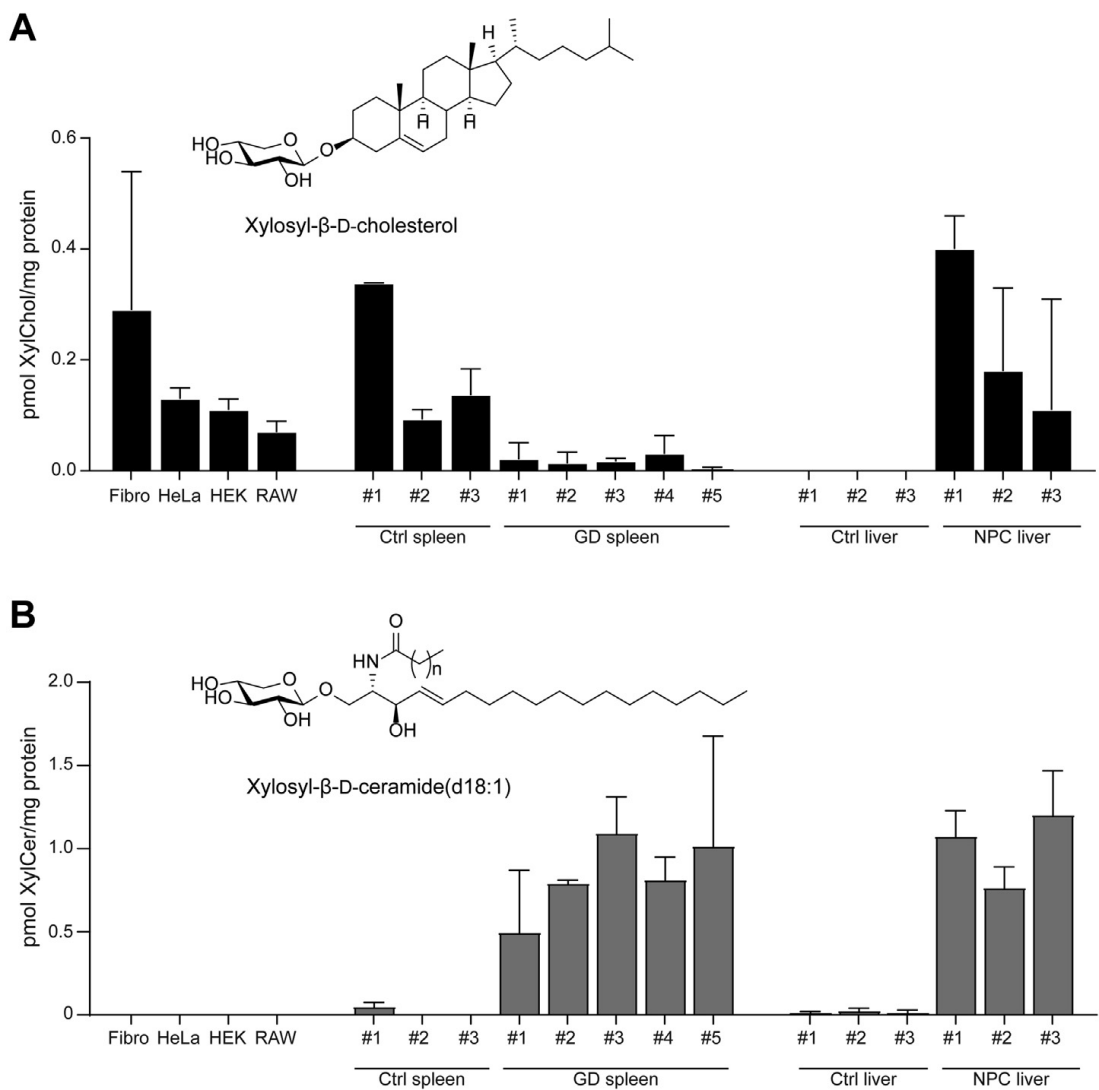
Levels of xylosylated cholesterol and xylosylated ceramide found in cells and organs. A: LC-MS/MS analysis of XylChol (A) and XylCer (B) occurrence in fibroblasts, HeLa, HEK293, and RAW264.7 cells; human control spleens; human GD spleens; mouse control livers; and mouse Npc1^−/−^ livers (n = 2 technical duplicates ± SD).

Next, the occurrence of XylChol in cells and tissues was investigated. Synthesized XylChol was used as the standard in LC-MS/MS quantification. Figure 4A shows the XylChol detection in lysates of 11 fibroblasts and HeLa, HEK293T, and RAW264.7 cells as well as in human spleen and mouse liver. The XylChol levels are relatively low as compared with those of HexChol (GlcChol and/or GalChol). In cells, XylChol levels are on average 130-fold lower than those of HexChol. In liver, relatively low amounts of XylChol were noted, but considerable XylChol levels were detected in liver of Npc1^−/−^ mice. The Npc1^−/−^ liver was earlier found to also contain high concentrations of GlcChol (10). Of note, reduced XylChol concentrations were observed in spleens of patients with type 1 GD.

### Xylosylated ceramide as a potential xylose donor for GBA

In our search for the endogenous xylose donor in the generation of XylChol, we investigated the possible occurrence of xylosylated ceramide (XylCer). For this purpose, an LC-MS/MS-based quantitation, based on microwave-assisted deacylation of XylCer to xylosylated sphingosine, was developed. We measured XylCer levels in the same materials previously used for XylChol measurements (Fig. 4B). The study revealed that XylCer is increased in GD spleen, whereas XylChol is reduced in the same tissue (Fig. 4A). Apparently, GBA does not degrade XylChol but rather synthesizes it. On the other hand, GBA can degrade XylCer. The observed increased levels of XylCer in NPC mouse liver can be likely ascribed to the secondary deficiency of GBA in this organ (10).

**Fig. 5:**
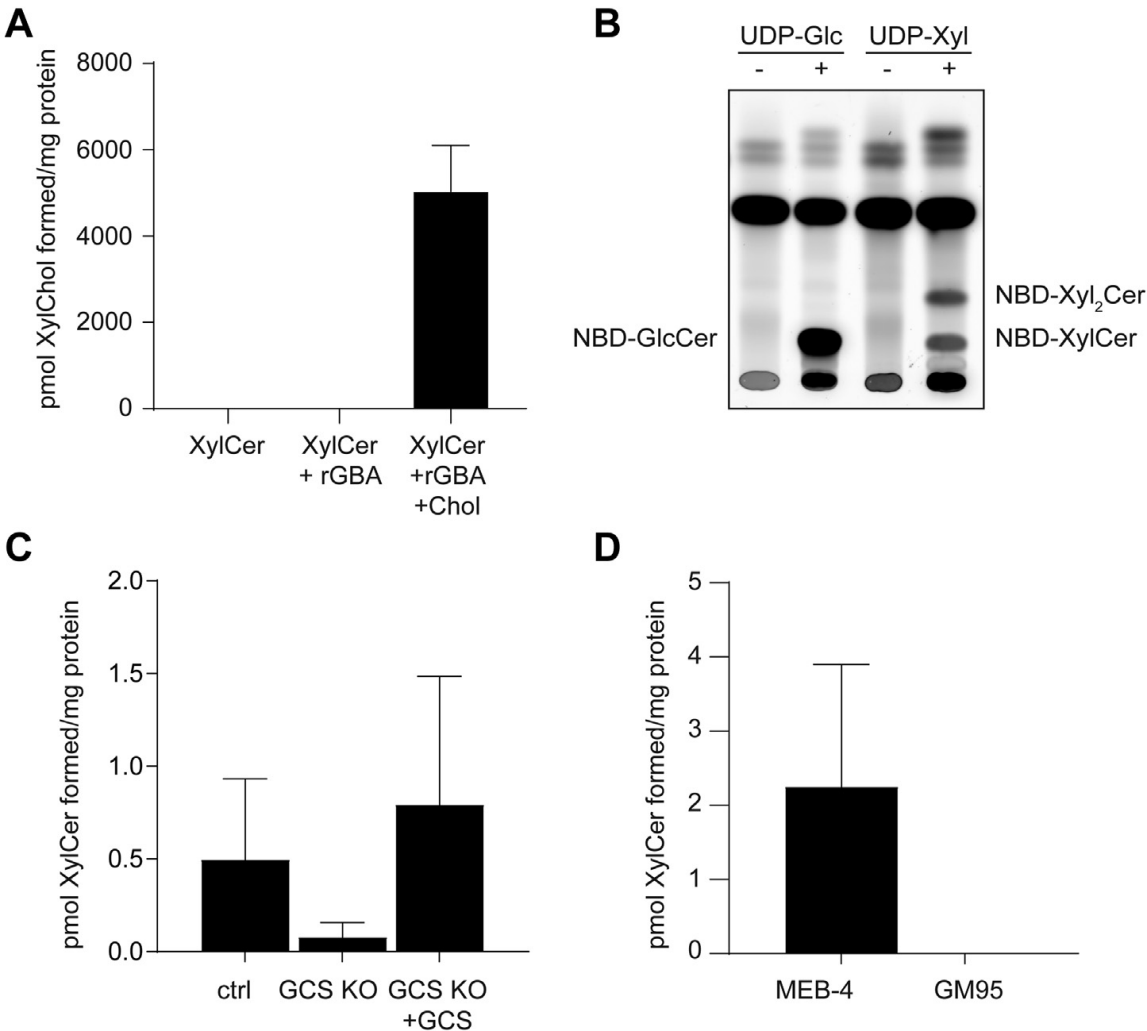
XylCer acts as a donor for GBA to transxylosylate cholesterol and can be synthesized by GCS. A: XylCer (formed by incubation of 4-MU-Xyl, Ceramide d18:1/18:1, and rhGBA at pH 5.2) was incubated with rhGBA and cholesterol at pH 5.2 for 18 h at 37°C. The figure depicts LC-MS/MS measurement of formed XylChol. Error bars represent standard deviation of technical duplicate. B: HPTLC analysis of formation of glycosylated NBD C6-ceramide by GCS with 0.2 mM NBD C6-ceramide and 20 mM UDP-Glc or UDP-Xyl as donor. C: LC-MS/MS analysis of formed xylosylated ceramide after 16 h incubation of cell lysates of control HeLa, HeLa GCS KO, or HeLa GCS KO with a reintegrated overexpression of GCS with UDP-Xyl and ceramide d18:1/18:1 (n = 2, mean ± SD). D: LC-MS/MS analysis of formed glycosylated ceramide after 16 h incubation of mouse fibroblast lysates with UDP-Xyl and ceramide d18:1/18:1 (n = 2, mean ± SD).

To test whether XylCer is a suitable sugar donor in GBA-mediated formation of XylChol, we enzymatically generated XylCer and then incubated it with 32 μM cholesterol and rhGBA. Abundant formation of XylChol was detected by LC-MS/MS (Fig. 5A), confirming that XylCer may act as a sugar donor in XylChol formation.

### Glucosylceramide synthase synthesizing xylosylated ceramide

Our discovery of XylCer stimulated a subsequent search for the XylCer-generating enzyme. As candidate, the enzyme GCS was tested. An in vitro assay with a lysate of HEK293T cells incubated with UDP-Xyl as donor and NBD C6-ceramide as acceptor led to the formation of NBD-XylCer and some NBDXyl_2_Cer (Fig. 5B). The same assay performed with ceramide d18:1/18:1 as acceptor resulted in the formation of XylCer as detected by LC-MS/MS (Fig. 5C). Lysates of cells lacking GCS were unable to generate XylCer. Expression of GCS in the GCS KO cells reinstalled their ability to produce XylCer (Fig. 5C). The ability of GCS to synthesise XylCer was further confirmed using lysates of the GCS-deficient GM95 cells and the parental GCS-competent MEB4 B16 cells incubated with UDP-Xyl and ceramide d18:1/18:1, where only the MEB4 cells showed formation of XylCer (Fig. 5D).

## Discussion

Here we report investigations into an intriguing catalytic feature of the lysosomal glucocerebrosidase, GBA. We noted first that beyond 4-MU-β-Glc, GBA also cleaves the substrate 4-MU-β-Xyl. Moreover, the enzyme uses both substrates as sugar donors in transglycosylation reactions with cholesterol as the acceptor. Next, we detected the generation of xylosylated cholesterol in living cells exposed to 4-MU-β-Xyl. Induction of lysosomal cholesterol accumulation in cells with U18666A increased the formation of xylosylated cholesterols, a reaction prevented by the inactivation of GBA with the irreversible inhibitors CBE or ME656 (16). Of special note, GBA even produces di-xylosyl-cholesterol using 4-MU-β-Xyl as the sugar donor in vivo as well as in vitro: this repetitive transglucosylation is not observed when 4-MU-β-Glc is used as the sugar donor (10).

The affinity of GBA for 4-MU-β-Glc as a substrate for hydrolysis exceeds that for 4-MU-β-Xyl. Likewise, XylChol is a much poorer substrate for hydrolysis by GBA than GlcChol. Following exposure of GBA to cholesterol and 4-MU-β-Xyl, the concentration of XylChol steadily builds up and it acts as acceptor in a second round of transxylosylation, rendering Xyl_2_Chol. Incubation of GBA and cholesterol with a mixture of 4-MU-β-Xyl and 4-MU-β-Glc leads to the formation of GlcXylChol (supplemental Table S3), further highlighting the suitability of XylChol as acceptor in transglycosylation by GBA. Of note, Aerts and coworkers earlier noted also a relative higher net transxylosylation than transglucosylation efficiency of a β-D-glucosidase from *Stachybotrys atra* (37), quite comparable with our findings with GBA. We docked GlcChol and XylChol in the crystal structure of human GBA. It appears that the CH_2_OH group in combination with N396 guides the substrate in an orientation that is optimal for hydrolysis. The lack of interaction between the CH_2_OH group and N396, as is the case with XylChol, probably results in a different orientation of the glycan moiety and hence affects the orientation and availability of the glucosidic bond for hydrolysis (supplemental Fig. S5). Obviously, crystallographic studies with soaked lipids in GBA crystals will be required to obtain conclusive data and further insight. Of note, Akiyama et al. recently reported that GBA is able to generate at a low rate galactosylated cholesterol (GalChol) from galactosylceramide and cholesterol (39). The enzyme, however, hardly hydrolyzes β-galactosides, including GalChol.

The possible physiological relevance of the observed transxylosylation by GBA warrants discussion. Using mass spectrometry, the presence of significant amounts of XylChol in the liver of Npc1^−/−^ mice could be demonstrated. XylChol was also detected in low quantities in cells, liver, and spleen. Of interest, in type 1 GD spleens XylChol levels were clearly reduced as compared with control spleens. This finding substantiates the notion that GBA is largely responsible for the formation of XylChol and poorly degrades it. In contrast, the enzyme hydrolyzes 4-MU-β-xyloside and GlcChol relatively much better.

The cytosol-facing membrane-bound β-glucosidase GBA2, shown earlier to potently transglucosylase cholesterol to generate GlcChol, has in contrast no significant activity toward β-xyloside substrates. Apparently the pendant CH_2_OH group in glucoside substrates contributes crucially to the interaction of substrate with GBA2. The importance of the presence of the additional CH_2_OH group in glucose also correlates with the much lower affinity of GBA2 for the inhibitor conduritol-B epoxide (with C5-hydroxymethyl group) compared with cyclophellitol (with C5-CH_2_OH group) (40). (34). The enzyme GBA2 also hydrolyzes β-galactosides and is able to generate GalChol via transgalactosylation (6, 39). In contrast to GBA2, the enzyme GBA3, a cytosolic broad-specificity glucosidase implicated in the metabolism of xenophobic glycosides (22), shows xylosidase and transxylosidase activity in vitro. The contribution of this enzyme in the metabolism of xylosides in tissues remains unclear.

Our discovery of several catalytic activities of GBA (i.e., β-glucosidase, β-xylosidase, transglucosylase, and transxylosylase activity) prompted us to explore the possibility that specific mutant forms of GBA that occur in patients with Gaucher disease may have selective abnormalities in one of these activities. Numerous amino acid substitutions in GBA are responsible for Gaucher disease, and several are sufficiently well characterized in their association with the clinical severity or phenotype in affected patients. For example, the heteroallelic presence of N370S GBA is protective for a neurological disease course in children and young adults, although it may be associated with Parkinsonism and dementia in later life (41). Analyzing patient fibroblasts that harbour the common mutations N370S, L444P, or the apparently unique D409H mutation in GBA that is associated with a unique clinical syndrome of cardiovascular disease did not indicate gross abnormalities in any of the catalytic activities, as tested with 4-MU-Glc and 4-MU-Xyl as substrates and cholesterol as the acceptor.

We were particularly interested in any abnormalities in the catalytic features of D409H GBA, since this mutation, when present in in homozygous form, is associated with extraordinary and unique clinical manifestations. The phenotype includes a chronic neurological phenotype with oculomotor apraxia and low-pressure hydrocephalus as well as the unusual feature of opacification of the cornea. However, the most striking feature in D409H homozygotes is Gaucher cell infiltration of the proximal aorta with calcification that involves the coronary arteries and left-sided cardiac valves (42, 43, 44). We observed a slightly reduced transglucosylase/glucosidase ratio for D409H GBA. Although the molecular basis of this phenotype remains unknown, we contend that careful study of the affected tissues obtained from patients who are affected for the presence of glucosylated metabolites offers perhaps the best opportunity for definitively solving the pathophysiology. The possibility that additional metabolites besides cholesterol are glucosylated via transglucosylation reactions is under active investigation.

In the course of our investigation a key question concerned the nature of physiological xyloside donors explaining the presence of XylChol. Several β-xylosidic compounds are known to be produced by plants and their uptake via food can “a priori not” be excluded (37, 38). Indeed, we did observe formation of XylChol in cells when incubated with cyanidin-3-O-β-D-xyloside, a ubiquitous compound in plums and berries. Thus, plant-derived β-xylosides might act as suitable donors for GBA-mediated formation of xylosylated sterols, but the quantitative importance of such reaction with an exogenous sugar donor is yet unclear. Theoretically, an alternative endogenous donor of β-D-xylosyl moieties might be β-D-xylosyl-peptides formed during the lysosomal degradation of proteoglycans.

Our investigation did reveal another, somewhat unexpected, endogenous β-xyloside: xylosylated ceramide. This unusual molecule has been previously reported to occur in the salt gland of the herring gull (45). We detected XylCer by mass spectrometry in various human and mouse tissues, although at very low concentrations compared with GlcCer; we moreover showed that XylCer serves as a sugar donor in the transxylosylation of cholesterol by GBA. Next, we observed that GCS is able to use UDP-Xyl to form XylCer, although with much lower affinity than UDP-Glc. XylCer was found to be elevated in the spleen of patients with Gaucher disease, a finding consistent with a role as a substrate for GBA and a sugar donor in transxylosylation mediated by the lysosomal enzyme. However, at the time of writing, the physiological relevance of the relatively minute amounts of XylCer is unclear and merits further investigation. Further investigation will also be required to establish to what extent exogenous β-xylosides of plant origin or endogenous β-xylosides (β-D-xylosyl-peptides or XylCer) serve as donors in the formation of XylChol.

Our findings regarding xylosylated lipids are summarized in the scheme. We observed that ceramide can be modified by the enzyme GCS to xylosyl-ceramide. This, or an exogenous β-xyloside, can act as donor in the formation of xylosyl-cholesterol by the enzyme GBA via a transglucosylation reaction. The formed xylosyl-cholesterol is poorly degraded and may act in the presence of large amounts of β-xyloside as acceptor in the further addition of xylosyl-moieties by GBA. The linkage of the subsequent xylosyl moieties is likely β-1,4, but β-1,2 or β-1,3 linkage cannot be excluded.

**Figure:**
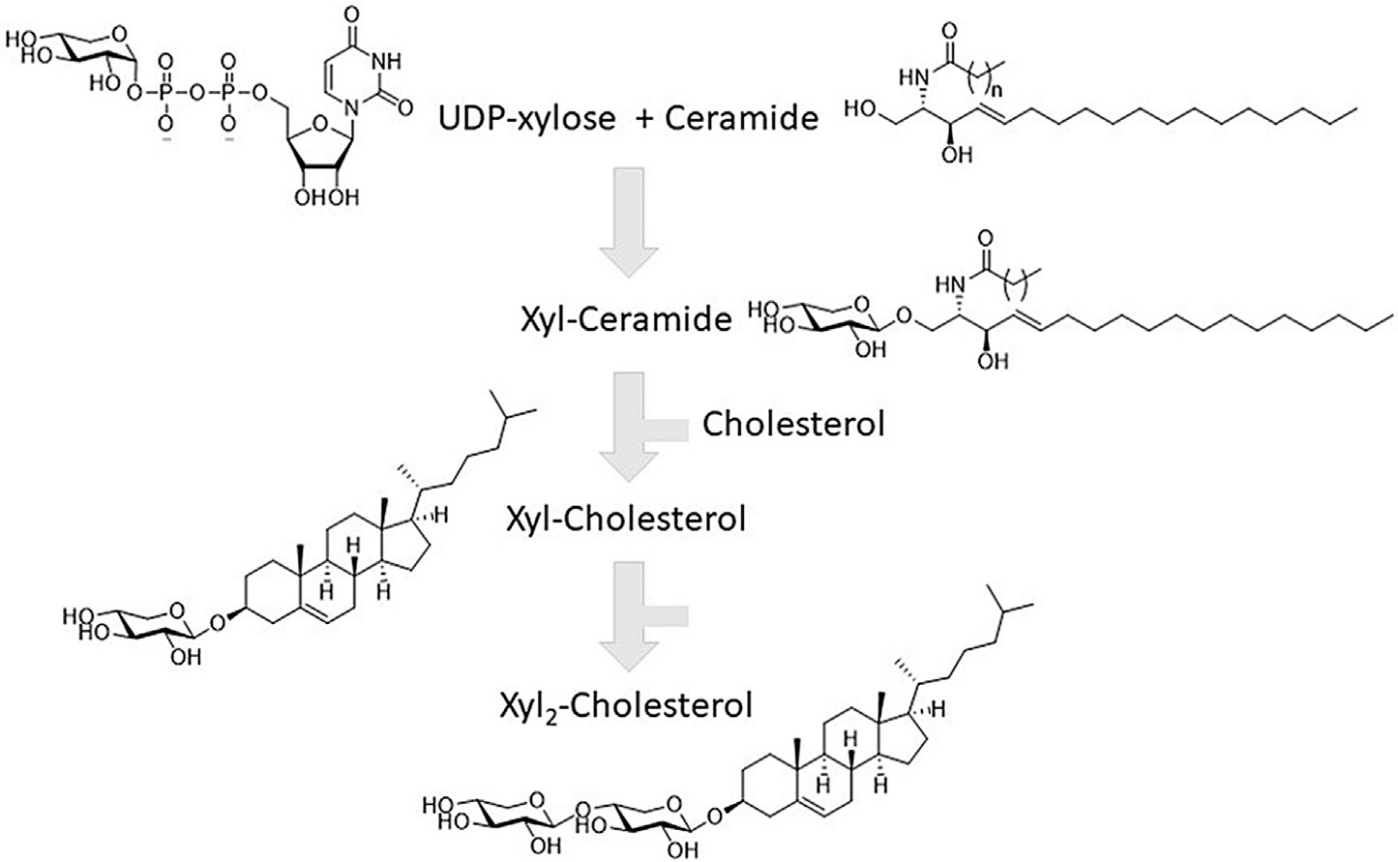

In conclusion, human GBA is more versatile in catalysis than hitherto considered. Investigation of the (patho)physiological relevance of various reactions catalyzed by GBA and xylosylated lipids is urgently needed to complete our understanding of the pathogenesis of Gaucher disease (7, 8) and other conditions for which abnormal GBA imposes a risk, such as multiple myeloma and α-synucleinopathies like Parkinsonism and Lewy-body dementia (46).

### Data availability

All the data are stored at the Department of Medical Biochemistry, Leiden University (The Netherlands). For further information, please contact corresponding author J. M. A. (j.m.f.g.gaerts@lic.leidenuniv.nl).

## Conflict of interest

The authors declare that they have no conflicts of interest with the contents of this article.
