# Supplementary material for: Human glucocerebrosidase mediates formation of xylosyl-cholesterol by β-xylosidase and transxylosidase reactions
Source: J Lipid Res. 2021 Jan 6;62:100018. doi: 10.1194/jlr.RA120001043 (PMC7903134; doi:10.1194/jlr.RA120001043)
Supplement: Supplemental data [file mmc1.pdf]

β-Xylosidase and transxylosidase reactions of human glucocerebrosidase

Daphne E.C. Boer[1*], Mina Mirzaian[1,a*], Maria J. Ferraz[1*], Kimberley C. Zwiers[1], Marc Hazeu[1], Merel V. Baks[1], Roelof Ottenhoff[2], André R.A. Marques[1,b], Rianne Meijer[1], Jonathan C.P. Roos[3], Timothy M. Cox[3], Rolf G. Boot[1], Navraj Pannu[4], Herman S. Overkleeft[5], Marta Artola[1], Johannes M. Aerts[1#].

Supplemental Materials and Methods

Synthesis of β-cholesteryl xyloside (β-Xyl-Chol)

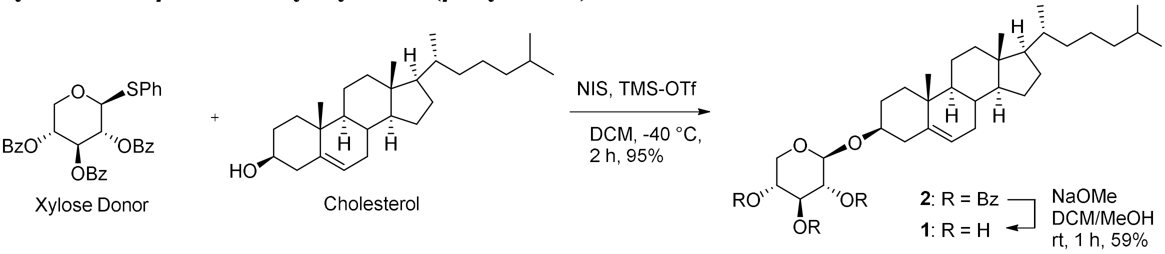

Starting materials, reagents and solvents were purchased as high-grade commercial products from Sigma-Aldrich and were used without further purification. Dichloromethane (DCM) stored over 4 Å molecular sieves, which were dried *in vacuo* before use. Reactions were monitored by analytical thin-layer chromatography (TLC) using Merck aluminium sheets pre-coated with silica gel 60 with detection by UV absorption (254 nm) and by spraying with a solution of $(NH_4)_6Mo_7O_{24}\cdot H_2O$ (25 g/L) and $(NH_4)_4Ce(SO_4)_4\cdot H_2O$ (10 g/L) in 10% sulfuric acid followed by charring at ~150 ˚C or by spraying with an aqueous solution of $KMnO_4$ (7%) and K_2CO_3 (2%) followed by charring at ~150 °C. Column chromatography was performed manually using either Baker or Screening Device silica gel 60 (0.04 - 0.063 mm), or with a Biotage Isolera™ flash purification system using silica gel cartridges (Screening devices SiliaSep HP, particle size 15-40 μm, 60A) in the indicated solvents. 1H NMR and 13C NMR spectra were recorded on Bruker AV-500 (500/125 MHz) spectrometer in the given solvent. Chemical shifts are given in ppm relative to the chloroform, methanol, or dimethylsulfoxide residual solvent peak or tetramethylsilane (TMS) as internal standard. The following abbreviations are used to describe peak patterns when appropriate: s (singlet), d (doublet), t (triplet), q (quartet), qt (quintet), m (multiplet), br (broad), ar (aromatic), app (apparent). LC/MS analysis was performed on A Waters Acquity TM TQD instrument. The instrument consisted of a UPLC system combined with a tandem quadrupole mass spectrometer as mass analyzer using a BEH C18 reversed-phase column (2.1 × 50 mm, particle size 1.7 μm; Waters Corporation), by applying an isocratic elution of mobile phases, 2-propanol:water 90:10 (v/v) containing 10 mM ammonium formate (eluent A) and methanol containing 10 mM ammonium formate (eluent B).

β-cholesteryl xylosyl benzoate 2.

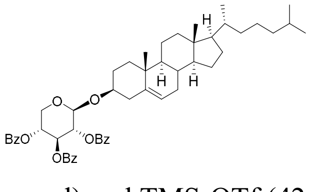

(2*S*,3*R*,4*S*,5*R*)-2-(phenylthio)tetrahydro-2*H*-pyran-3,4,5-triyl tribenzoate donor1 (111 mg, 0.20 mmol) and cholesterol (62 mg, 167 mmol) were co-evaporated in toluene (2x) and re-dissolved in 2 mL of DCM. 4Å molecular sieves were added and the mixture was stirred for 30 min at room temperature. Then the mixture was cooled to -40 °C and NIS (45 mg, 0.20 mmol) and TMS-OTf (42 μL, 0.23 mmol) were added. After stirring for 2 h at -40 °C the reaction was quenched with Et_3N and warm up to room temperature. The mixture was diluted with DCM (30 mL) and aqueous 10% $Na_2S_2O_3$ (20 mL). The extracted organic phase was then washed with brine, dried and purified by silica gel column chromatography (from pentane to pentane:EtOAc 9:1) to afford the protected β-cholesteryl xyloside **2** in 95% yield. ^1H NMR (500 MHz, $CDCl_3$): *δ* 7.99 (ddd, *J* = 7.1, 4.1, 1.2 Hz, 5H), 7.55 – 7.47 (m, 3H), 7.36 (q, *J* = 8.0 Hz, 6H), 5.76 (t, *J* = 7.4 Hz, 1H), 5.34 (dd, *J* = 7.5, 5.6 Hz, 1H), 5.29 – 5.27 (m, 1H), 4.95 (d, *J* = 5.6 Hz, 1H), 4.44 (dd, *J* = 12.1, 4.4 Hz, 1H), 3.68 (dd, *J* = 12.1, 7.3 Hz, 1H), 3.57 (tt, *J* = 11.3, 4.6 Hz, 1H), 2.46 – 2.33 (m, 1H), 2.27 (ddd, *J* = 13.3, 4.9, 2.2 Hz, 1H), 2.20 – 2.11 (m, 1H), 2.06 – 1.90 (m, 2H), 1.90 – 1.76 (m, 3H), 1.73 – 1.47 (m, 8H), 1.47 – 1.19

(m, 6H), 1.19 – 1.02 (m, 6H), 1.02 – 0.97 (m, 2H), 0.95 (s, 3H), 0.91 (d, *J* = 6.6 Hz, 3H), 0.87 (d, *J* = 2.2 Hz, 3H), 0.85 (d, *J* = 2.2 Hz, 3H), 0.66 (s, 3H). ^{13}C NMR (126 MHz, $CDCl_3$) *δ* 165.7, 165.6, 165.4, 140.5, 133.5, 133.3, 130.0, 129.6, 129.4, 129.4, 128.5, 122.2, 98.8, 79.0, 77.4, 77.2, 76.9, 70.9, 70.7, 69.5, 61.5, 56.9, 56.3, 50.3, 42.5, 39.9, 39.7, 38.8, 37.4, 36.9, 36.3, 35.9, 32.1, 32.0, 29.9, 29.7, 28.4, 28.3, 28.2, 24.4, 24.0, 23.0, 22.7, 21.2, 19.5, 18.9, 12.0.

β-cholesteryl xyloside 1.

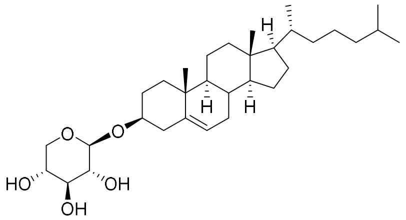

Intermediate **2** (63 mg, 0.076 mmol) was dissolved in a mixture of DCM/MeOH (1:2, v/v, 3 mL) and NaOMe (5.4 M in MeOH, 7.6 µL). After stirring for 1 h the reaction mixture was neutralized by addition of $Et_3N·HCl$ and purified by silica gel column chromatography (from DCM to DCM:MeOH 9:1), yielding the title compound cholesteryl-D-β-Xylopyranoside (β-Xyl-Chol) **1** as a white solid in 59% yield. ^{1}H NMR (500 MHz, $CDCl_3$): *δ* 5.37 (d, *J* = 5.1 Hz, 1H), 4.50 (d, *J* = 6.1 Hz, 1H), 4.05 (dd, *J* = 11.9, 4.4 Hz, 1H), 3.75 (dq, *J* = 8.0, 4.0 Hz, 1H), 3.65 – 3.53 (m, 2H), 3.47 – 3.40 (m, 1H), 3.36 (dd, *J* = 11.9, 8.1 Hz, 1H), 2.94 (s, 1H), 2.64 (d, *J* = 4.1 Hz, 1H), 2.42 – 2.34 (m, 2H), 2.23 (t, *J* = 12.5 Hz, 1H), 2.04 – 1.95 (m, 2H), 1.91 – 1.81 (m, 2H), 1.64 – 1.43 (m, 10H), 1.36 – 1.23 (m, 6H), 1.16 – 1.04 (m, 6H), 1.00 (s, 3H), 0.91 (d, *J* = 6.5 Hz, 3H), 0.87 (d, *J* = 2.3 Hz, 3H), 0.86 (d, *J* = 2.4 Hz, 3H), 0.67 (s, 3H). ^{13}C NMR (126 MHz, $CDCl_3$) *δ* 140.2, 122.5, 100.8, 78.7, 74.6, 72.5, 69.9, 64.3, 56.9, 56.3, 50.3, 42.5, 39.9, 39.7, 38.8, 37.4, 36.9, 36.3, 35.9, 32.1, 32.0, 29.8, 28.4, 28.2, 24.4, 24.0, 23.0, 22.7, 21.2, 19.5, 18.9, 12.0. LC-MS/MS: calcd. for $[C_{32}H_{54}O_5+NH_3]^+$ 518.8; found 536.7.

^{1}H-NMR and ^{13}C-NMR spectra of **2** in $CDCl_3$

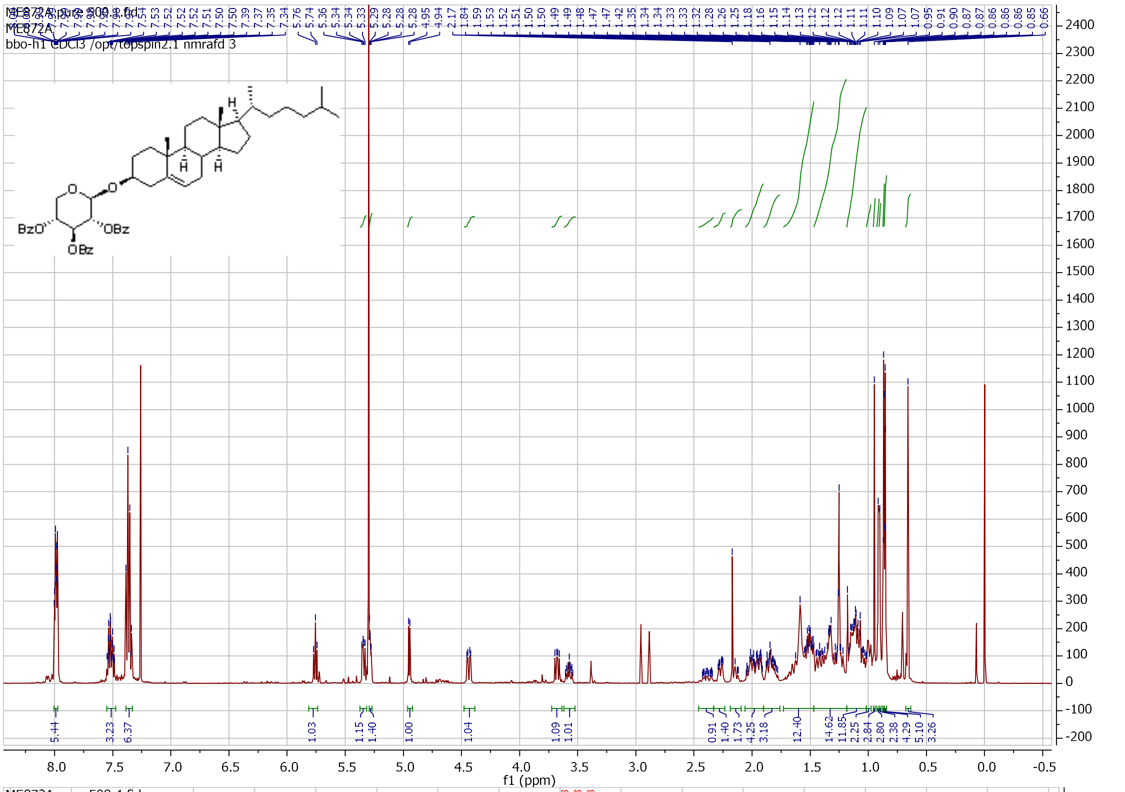

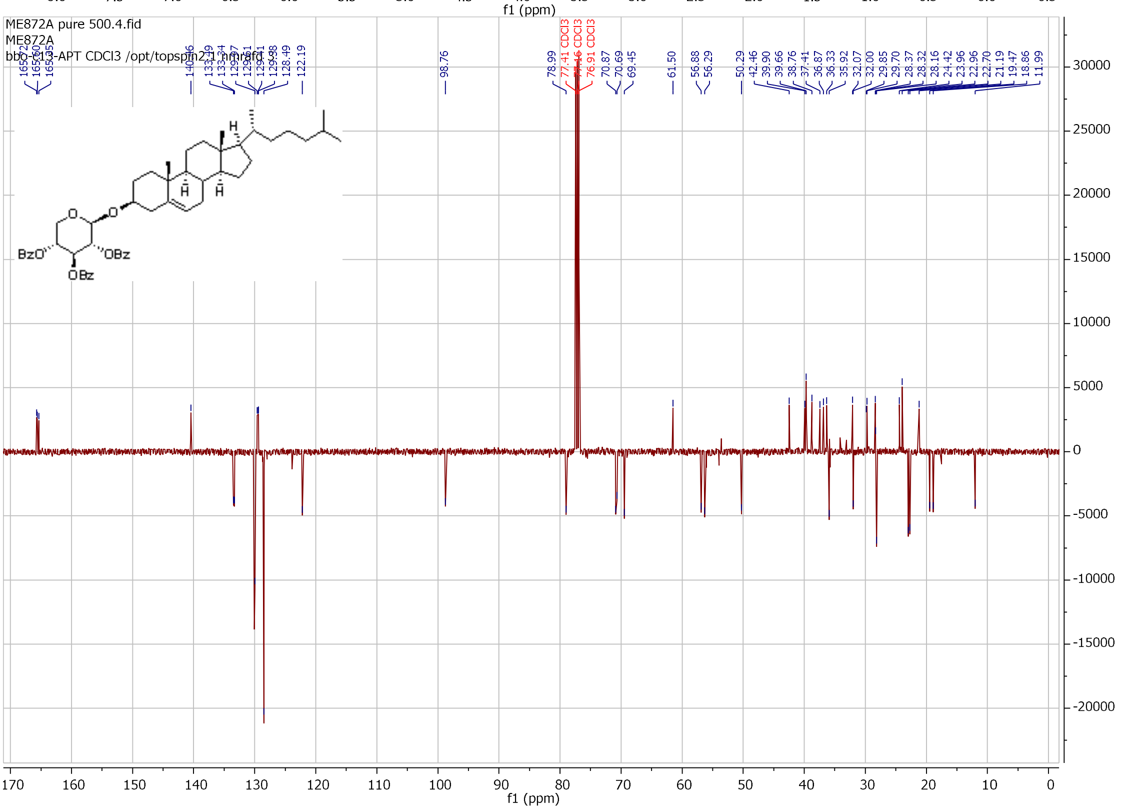

^{1}H-NMR and ^{13}C-NMR spectra of **1** in $CDCl_3$

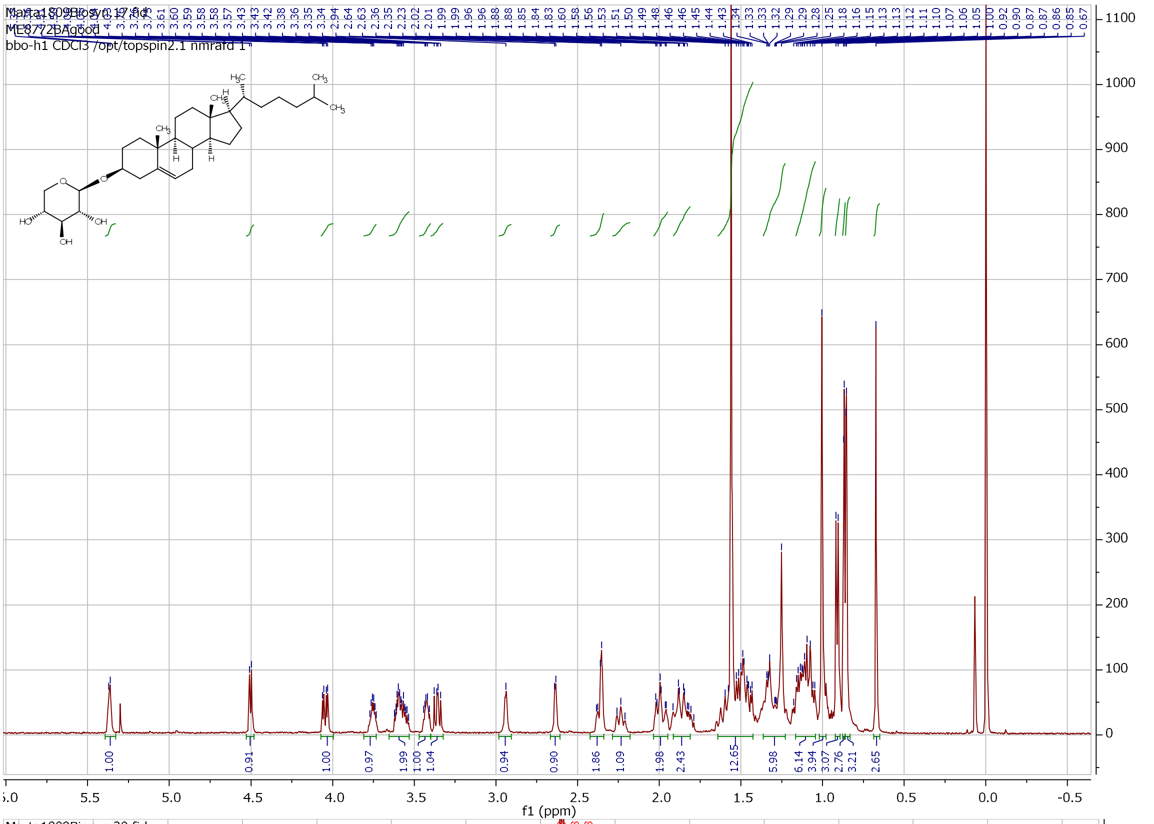

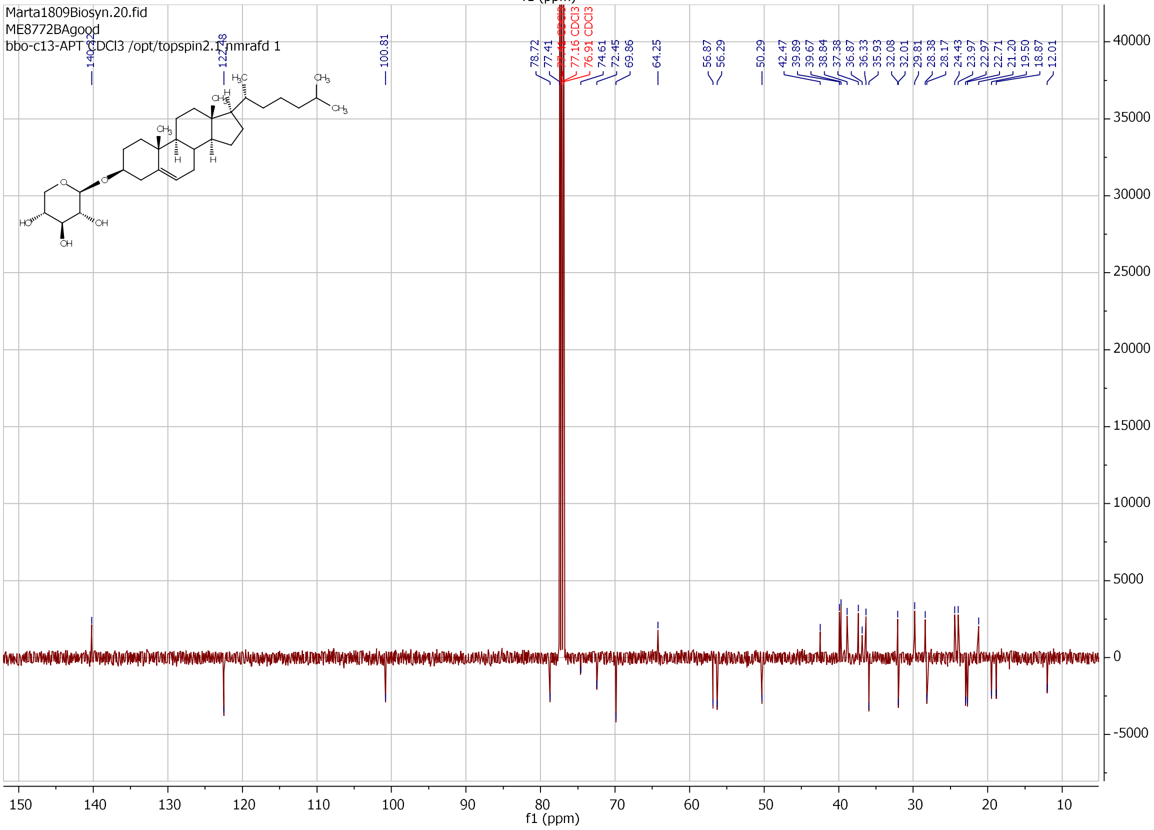

Supplemental figures

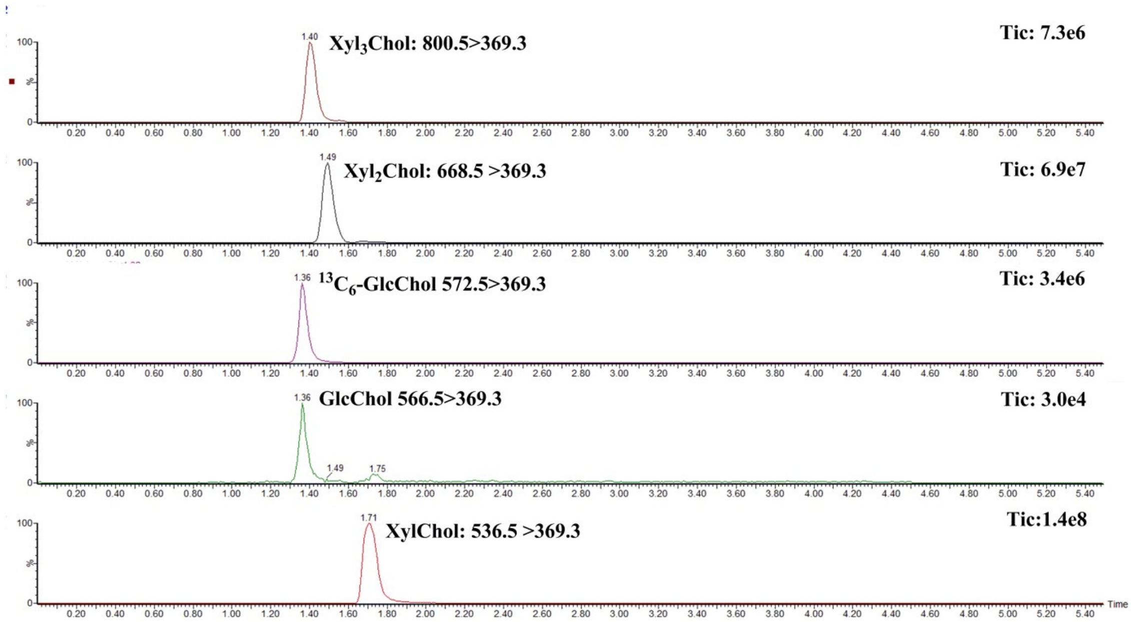

Supplemental Figure S1: Chromatogram of XylChol, Xyl$_2$Chol, Xyl$_3$Chol, GlcChol and $^{13}C_6$-GlcChol.

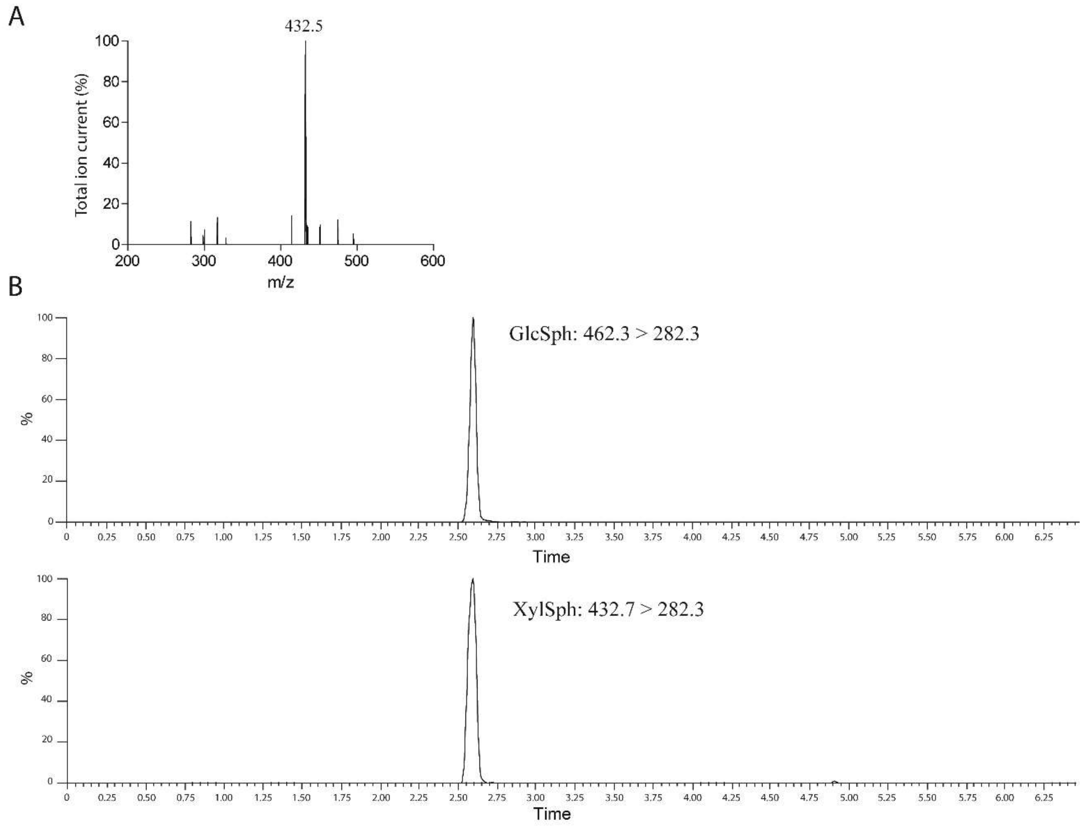

Supplemental Figure S2: A. MS/MS Fragmentation spectrum of parent scan XylSph (parent of 282.3) B. Chromatogram GlcSph and XylSph.

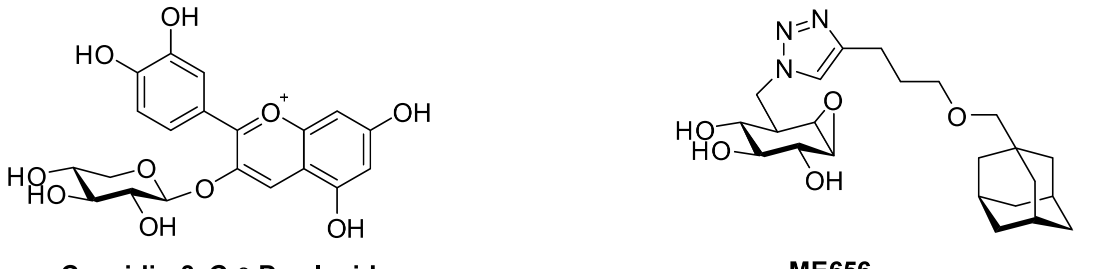

Supplemental Figure S3: Chemical structures of cyanidin-3-*O*-β-D-xyloside and ME656 GBA irreversible inhibitor.

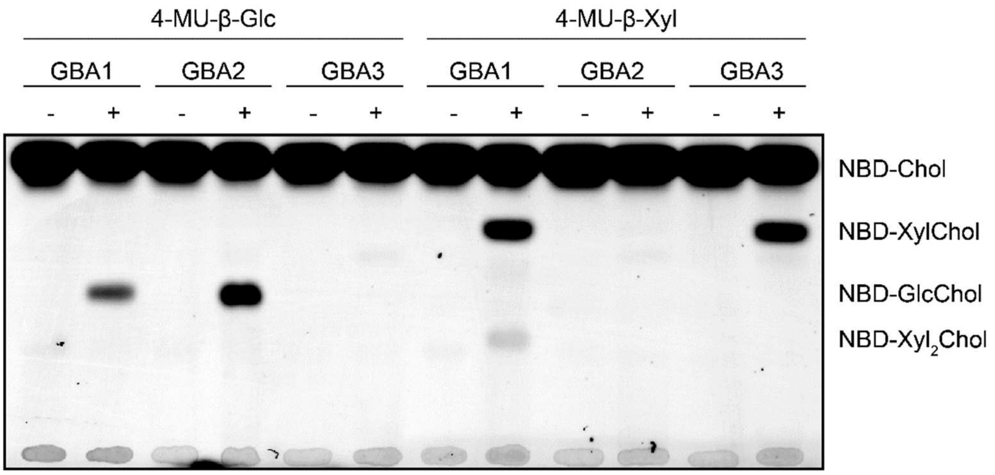

Supplemental Figure S4: Lack of transxylosylation by GBA2. HPTLC analysis of formation of glycosylated 25-NBD-cholesterol by β-glucosidases with 4-MU-β-Xyl and 4-MU-β-Glc as donor. Enzymes: recombinant rhGBA; lysate of HEK293 cells overexpressing GBA2; lysate of HEK293 cells overexpressing GBA3. Incubation for 16 hours with (+) or without (-) enzyme preparation.

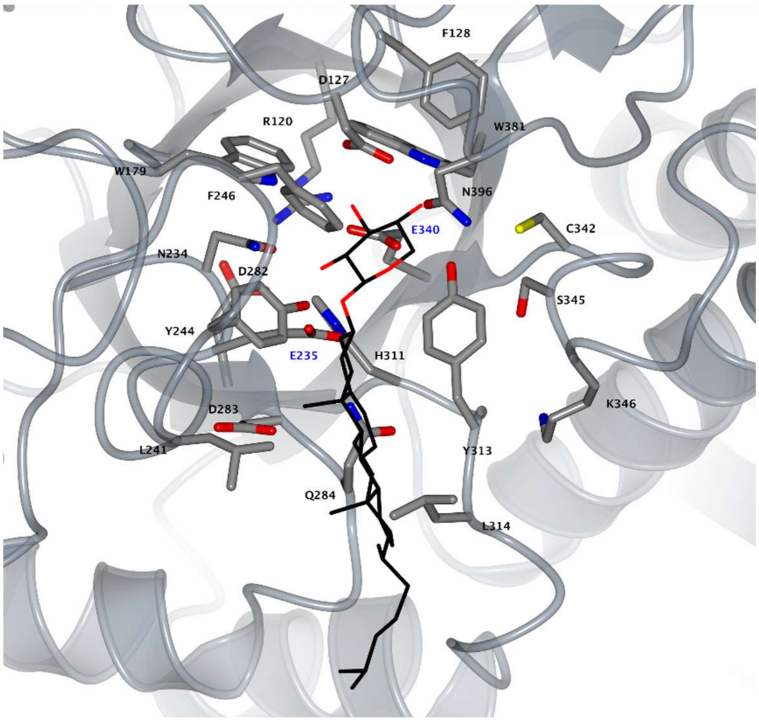

Supplemental Figure S5: Docking of XylChol in GBA. XylChol was docked in GBA crystal structure (PDB 2V3D), with carbon (black) and oxygen (red) atoms indicated. GBA is shown as grey ribbons and residues lining Xylchol, together with catalytic residues Glu235 and Glu340, are also visualized.

Supplemental tables

Supplemental Table S1. Kinetic parameters rhGBA

**rhGBA**	**4MU-β-Glc**	**4MU-β-Xyl**
K_m (mM)	0.76 ± 0.06	5.24 ± 1.04
V_{max} (nmol/h.mg)	1.23 ± 0.03 x 10^6	1.88 ± 0.3 x 10^5
K_{cat}/K_m (mM/s^{-1})	25.03	0.55
Apparent IC_{50} cyclophellitol (µM)	0.085 ± 0.002	0.061 ± 0.002
Apparent IC_{50} D-xylo-cyclophellitol (µM)	10.16 ± 1.03	6.41 ± 0.47

Supplemental Table S2. MS/MS instrument parameters.

Mass spectrometer	Xevo-TQ-S micro (Waters)
Ionization mode	ESI^+
Capillary voltage	3.50 kV
Source temperature	150 °C
Desolvation temperature	450 °C
Cone gas flow	50 L/h
Desolvation gas flow	950 L/h

Compound	Parent (m/z)	Daughter (m/z)	Cone voltage (V)	Collision energy (V)	Retention time (min)
GlcChol	566.5	369.3	20	15	1.34
^{13}C-GlcChol	572.5	369.3	20	15	1.34
XylChol	536.5	369.3	20	15	1.69
Xyl_2Chol	668.5	369.3	20	15	1.47
Xyl_3Chol	800.5	369.3	20	15	1.38
GlcSph	462.3	282.3	30	20	3.23
XylSph	432.7	282.3	25	15	3.25
C17-Sphinganine	288.3	270.3	20	15	3.26

Supplemental Table S3. Formation of hybrid GlcXylChol following incubation of GBA with 1:1 mixture of 4-MU-β-Xyl and 4MU-β-Glc.

**MRM transitions**	**4-MU-Xyl (3h) 4-MU-Glc (1h)**	**pmol/mL**
535.5 > 369.3	XylChol	631.56
566.5> 369.3	GlcChol	293.20
668.5> 369.3	Xyl_2Chol	76.34
698.5> 369.3	GlcXylChol	10.58
800.5> 369.3	Xyl_3Chol	3.34
